# Enhancing reporting through structure: a before and after study on the effectiveness of SPIRIT-based templates to improve the completeness of reporting of randomized controlled trial protocols

**DOI:** 10.1186/s41073-024-00147-7

**Published:** 2024-05-31

**Authors:** David Blanco, Márcio Vinícius Fagundes Donadio, Aïda Cadellans-Arróniz

**Affiliations:** 1 Department of Physiotherapy, Universitat Internacional de Catalunya, C/Josep Trueta S/N., Sant Cugat del Vallès, 08195 Barcelona, Spain; 2 Pontifícia Universidade Católica Do Rio Grande Do Sul (PUCRS), Porto Alegre, Brazil

**Keywords:** Meta-research, Randomized controlled trial, Reporting guidelines, Reporting quality, Clinical trial protocol

## Abstract

**Background:**

Despite the improvements in the completeness of reporting of randomized trial protocols after the publication of the Standard Protocol Items: Recommendations for Interventional Trial (SPIRIT) guidelines, many items remain poorly reported. This study aimed to assess the effectiveness of using SPIRIT-tailored templates for trial protocols to improve the completeness of reporting of the protocols that master’s students write as part of their master’s theses.

**Methods:**

Before and after experimental study performed at the University Master’s Degree in Orthopaedic Manual Physiotherapy of the Universitat Internacional de Catalunya (Barcelona, Spain). While students in the post-intervention period were instructed to use a trial protocol template that was tailored to SPIRIT, students in the pre-intervention period did not use the template. Primary outcome: Difference between the pre- and post-intervention periods in the mean number of adequately reported items (0–10 scale). The outcomes were evaluated independently and in duplicate by two blinded assessors. Students and their supervisors were not aware that they were part of a research project. For the statistical analysis, we used a generalized linear regression model (dependent variable: number of adequately reported items in the protocol; independent variables: intervention period, call, language).

**Results:**

Thirty-four trial protocols were included (17, pre-intervention; 17, post-intervention). Protocols produced during the post-intervention period (mean: 8.24; SD: 1.52) were more completely reported than those produced during the pre-intervention period (mean: 6.35; SD: 1.80); adjusted difference: 1.79 (95% CI: 0.58 to 3.00).

**Conclusions:**

SPIRIT-based templates could be used to improve the completeness of reporting of randomized trial protocols.

**Supplementary Information:**

The online version contains supplementary material available at 10.1186/s41073-024-00147-7.

## Introduction

The Standard Protocol Items: Recommendations for Interventional Trials (SPIRIT) guidelines were published in 2013 to improve the completeness of reporting of randomized controlled trial (RCT) protocols [1]. Despite the improvements in the completeness of reporting of RCT protocols after the publication of the SPIRIT guidelines, many items remain poorly reported [2, 3]. Adherence to other common reporting guidelines, such as the Consolidated Standards of Reporting Trials (CONSORT) [4] or the Preferred Reporting Items for Systematic Reviews and Meta-Analyses (PRISMA) [5], is also suboptimal [6].

Currently, the prevailing approach adopted by biomedical journals to increase completeness of reporting across various reporting guidelines is to force authors to submit a checklist from the relevant guideline [7]. Nevertheless, it has been shown that this policy does not have a significant impact, as completed checklists are often overlooked by editors and reviewers [8, 9]. In recent years, different interventions to improve adherence to reporting guidelines have been proposed [7]. Although the effectiveness of most of these has not been examined (and even fewer with RCTs), some have shown promising results [10–15]. A recent RCT has shown the benefits of involving a CONSORT expert in the peer review process [12]. However, asking standard peer reviewers to check specific reporting guideline items has been shown not to improve adherence to CONSORT and SPIRIT [16]. Some authors have argued that researchers need additional support during the initial stages of the research process, such as the manuscript writing stage [13]. For this reason, Barnes et al. carried out an RCT that tested the effect of using an online writing aid tool for writing RCT reports and showed its benefits [13]. Also, adapting the traditional Introduction, Methods, Results, and Discussion (IMRaD) structure of RCTs to the requirements of CONSORT [14] or reporting the results of RCTs in a tabular way [15] have been associated with an increase in completeness of reporting. Consistent with this approach, the journal *Trials* started offering the option to submit SPIRIT-tailored protocols [17, 18] to be considered for publication. Although this is a strategy with considerable potential and has no cost to authors or journals, it has still not been empirically evaluated.

Previous research has shown that reporting guidelines could be used as educational tools by undergraduate, master’s or PhD students to develop more complete and transparent study protocols [19]. Due to the importance of research-based educational interventions in helping biomedical students acquire research-related competencies [20] and the promising effect of these interventions [21], using reporting guidelines as educational tools has great potential for improving the quality of study protocols. To our knowledge, no study has evaluated this topic to date.

For these reasons, this study aimed to assess the effectiveness of using templates for RCT protocols tailored to SPIRIT guidelines to improve the completeness of the RCT protocols that master’s students write as part of their master’s theses.

## Methods

### Study design and setting

This was before and after experimental study. This study type is suitable for determining the effects of a certain intervention by comparing the outcomes of study participants (who can be the same or different people) investigated before this intervention with those measured afterward [22]. The study was performed in the context of the University Master’s Degree in Orthopaedic Manual Physiotherapy of the Universitat Internacional de Catalunya (Barcelona, Spain).

The protocol of this study is available in a public repository [23].

### Eligibility criteria

We included protocols for RCTs developed by master’s students as part of their master’s theses. These protocols were written in English or Spanish and were eligible if they were submitted in the 2020–2021 course (before the implementation of the intervention) or in the 2021–2022 course (after the implementation). Other types of studies were excluded.

### Interventions

The intervention consisted of two steps. First, the lead investigator (DB) delivered an RCT protocol template (see Additional file 1) in English and Spanish to the master’s students. This was done via email and via Moodle, the learning management system used in the master’s program. The template was tailored to the SPIRIT guidelines [1], meaning that it contained SPIRIT items as subheadings within the Introduction, Methods, Results, and Discussion (IMRaD) structure of the protocol. Additionally, a short explanation of each item was included, and a full explanation and examples of adequate reporting for each item can be found in the SPIRIT Explanation & Elaboration document [1]. To develop the template in Spanish, we used the official Spanish translation of SPIRIT [24]. In the second step, these SPIRIT items were reviewed during a 3-h session that was part of the Research Methodology subject of the master’s program. Students were instructed to use the template, either in English or in Spanish, when writing up their master’s theses. Although the use of the template was not compulsory, it was recommended that the participants at least adhere to the proposed subheadings. Some SPIRIT items related to ethics and data analysis (i.e., access to data or plans to promote participant retention) were not included in the template because they exceeded the expectations of master’s theses.

For the RCT protocols that were carried out before the implementation of the intervention, that is, in the 2020–2021 academic course, students were not instructed to use any template related to the SPIRIT guidelines and had to include less specific subheadings (e.g., background, objectives, study type, participants, variables, interventions, statistical analysis, ethical considerations, and study timeline). As in the post-intervention period, the SPIRIT items that were included in the templates were reviewed during a 3-h session.

This intervention was implemented at no cost and did not cause any disruption to the normal operating procedures of the master’s program.

### Outcomes and data collection methods

- *Primary outcome:* Difference between the pre- and post-intervention periods in the mean number of adequately reported items in the RCT protocol among 10 selected SPIRIT items (0–10 scale).
- *Secondary outcome:* Proportion of manuscripts in pre- and post-intervention periods where each item was adequately reported.

The study outcomes were independently evaluated in duplicate by two blinded outcome assessors (MD, AC) who were familiar with the methodology and reporting of the RCT protocols. To train the outcome assessors and to ensure that their evaluations were as consistent as possible, they appraised two random RCT protocols and discussed their disagreements. For the final evaluations, discrepancies among outcome assessors were also discussed until a consensus was reached. The outcome evaluation took place between June and September 2023.

To determine what information is expected to be reported for each item, we relied on the SPIRIT Explanation and Elaboration document [1]. A SPIRIT item was considered adequately reported if all subparts of the item were adequately reported according to the SPIRIT guidelines (e.g., for SPIRIT item 12a: A) completely prespecified primary and secondary outcomes, B) how each of these outcomes is assessed, and C) when each of these outcomes is assessed). Further details about how certain SPIRIT items were assessed can be found in Additional file 2.

We evaluated the reporting of 10 core SPIRIT items from the Methods section; these items are usually poorly reported [25]. Table 1 describes each of these items.

**Table 1:**
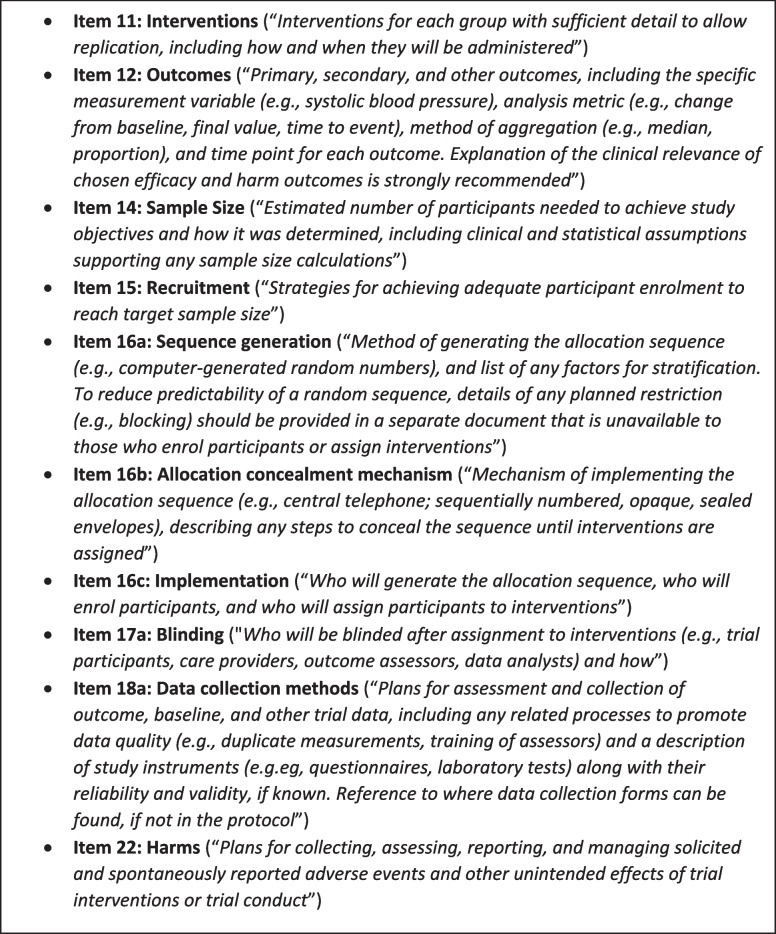
SPIRIT items considered

### Sample size

We used the function *pwr.t.test* (underlying test: t test) within the package “pwr” in R Statistical Software [26] to perform the sample size calculation. Based on the findings of a previous study [12], whose results for CONSORT guidelines we aimed to replicate in this study, we estimated a between-group difference of 1.43 points (0–8 scale) and a common SD of 1.45. Assuming an alpha risk of 0.05 and a beta risk of 0.2, the sample size needed for this study was 34 manuscripts (17 in the pre-intervention period and 17 in the post-intervention period).

### Recruitment

The lead investigator was granted access (see “Informed Consent and Materials”) to all master’s theses produced during the 2020–2021 and 2021–2022 academic courses in the master’s program mentioned above. These theses were filtered based on the eligibility criteria.

### Blinding

Students and their supervisors were not aware that their RCT protocols were part of a research project. The outcome evaluators were blinded to whether the protocols were written in the pre- or post-intervention period. To protect their blinding, the lead investigator removed the front page of the protocols, the references, and any other information that could threaten blinding (i.e., any temporal reference or participant personal data).

### Statistical methods

We used R Statistical Software [26] to perform the statistical analysis. First, we calculated descriptive statistics for each study period, including the percentage of protocols that were written in each language (Spanish or English), the submission attempt (first-sitting vs first retake), the type of intervention proposed (pharmacologic or nonpharmacologic), and the mean (SD) baseline and final values for the primary outcome. Second, we used a generalized linear regression model in which the dependent variable was the number of adequately reported items in the RCT protocol and the independent variables were the academic year (before or after the intervention), the language, and the submission attempt. We tested the model assumptions (linearity, normality, homoscedasticity, and absence of collinearity). Based on this model, we observed the effect size of the intervention. We calculated the 95% confidence interval (95% CI) using bootstrapping. All R codes are shown in Additional file 3.

The interrater agreement was analysed using percentage agreement and Cohen's kappa coefficient.

## Results

Among the 2020–2021 and 2021–2022 academic courses, 47 master’s theses were produced (pre-intervention period: 27; post-intervention period: 20). We excluded 7 of these (pre-intervention period: 5; post-intervention period: 2) because they were not RCT protocols. Among the 40 candidates, we chose a random sample of 34 RCT protocols (17 per period). All included protocols have been made available in a public repository [23].

Most protocols (*n* = 32, 94%) were written in Spanish, and only 2 (6%) were written in English. Most of them (*n* = 25, 74%) were submitted in the first sitting (Table 2). All the manuscripts described nonpharmacological interventions in the field of physiotherapy (manual therapy, instrument-assisted therapy, or therapeutic exercise). No protocol in the post-intervention was elaborated by a student who had failed the subject in the pre-intervention period and who therefore had to retake it in the post-intervention period. The baseline characteristics of the protocols in the two study periods were similar (Table 2).

**Table 2:** Characteristics of the RCT protocols

		**Pre-intervention (** ***n*** ** = 17)**	**Post-intervention (** ***n*** ** = 12)**
Language	Spanish	16 (94%)	16 (94%)
	English	1 (6%)	1 (6%)
Call	1st (July)	12 (71%)	13 (76%)
	2nd (September)	5 (29%)	4 (24%)
Type of intervention	Pharmacologic	0 (%)	0 (0%)
	Nonpharmacologic	17 (100%)	17 (100%)

The outcome assessors initially agreed in the evaluation of 86.47% of the items (296 of 340), and the interrater agreement was moderate (κ = 0.66). In the second step, all disagreements were resolved by consensus. The dataset that contains the duplicate outcome assessment can be accessed in a public repository [23].

### Outcomes

#### Primary outcome

The RCT protocols that were produced during the post-intervention period were more completely reported than those produced during the pre-intervention period: post-intervention (mean: 8.24; SD: 1.52) versus pre-intervention (mean: 6.35; SD: 1.80). After adjusting for the other relevant covariates, the mean difference in scores between the two periods was 1.79 (95% CI = 0.58 to 3.00) favoring the post-intervention period. Table 3 shows these results.

**Table 3:** Completeness of reporting scores in the pre- and post-intervention periods

**Outcome**	**Pre-intervention period (** ***n*** ** = 17)** Mean (SD)	**Post-intervention period (** ***n*** ** = 17)** Mean (SD)	**Adjusted difference** (95% CI)
Completeness of reporting score (0 to 10 scale)	6.35 (1.80)	8.24 (1.52)	1.79 (0.75 to 3.01)

#### Secondary outcome

**Fig. 1:**
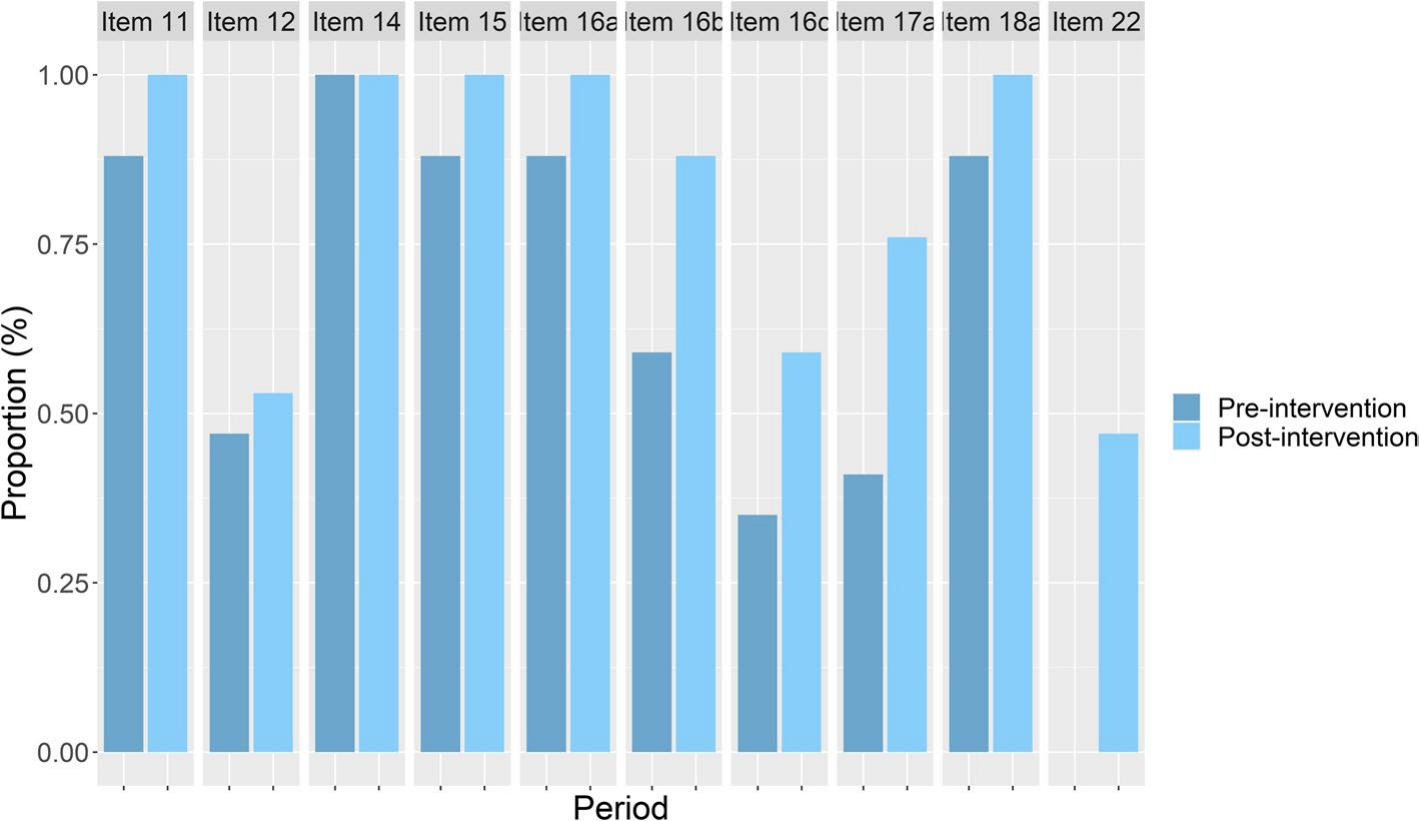
Proportion of RCT protocols (*n* = 34) in which each SPIRIT item was adequately reported. Legend: Dark blue: pre-intervention; Light blue: post-intervention

Figure 1 displays the proportions of manuscripts from each period where each SPIRIT item was adequately reported. Except for item 14 (sample size), which was adequately reported in all the included studies, all the items were more adequately reported in the post-intervention period. We observed the main differences favoring the post-intervention period in items 16b (allocation concealment mechanism), 16c (implementation), 17a (blinding), and 22 (Harms). Item 22 (Harms) was never properly reported during the pre-intervention period, while 47% (8 of 17) of the manuscripts from the post-intervention period reported it well.

## Discussion

This study showed the beneficial effect of using templates for RCT protocols tailored to the SPIRIT guidelines on the completeness of reporting of RCT protocols developed by master’s students. Specifically, 8.24 out of the 10 core SPIRIT items were reported to be used in RCT protocols where the templates were used; this number represents 1.79 items more (0–10 scale, 95% CI 0.90 to 2.85) than in protocols where the templates were not used. We observed the greatest differences for items 16b (allocation concealment mechanism), 16c (implementation), 17a (blinding), and 22 (harms).

Adherence to SPIRIT guidelines in the biomedical literature has improved over the time but is still low [2]. Previous studies have shown that RCT protocols adequately report on average 56.7% items of the whole checklist [2] and 45.6% of 10 core items [3]. In our study, protocols in the pre-intervention period adequately reported a slightly better percentage of the items (63.5%), which could be due to the 3-h instruction on the SPIRIT items that was delivered.

Our findings are in line with those of previous studies focused on CONSORT guidelines that show that the most successful strategies for improving adherence are those focused on helping authors at the writing stage of the manuscript and those involving reporting guideline experts in the peer review process. Barnes et al. performed an RCT in which RCT manuscripts were developed using the CONSORT-based online writing aid tool (COBWEB) reported an average of 2.1 CONSORT items (0–10 scale, 95% CI 1.5 to 2.7) more than did those that did not use that tool [13]. In the context of a dentistry journal, a cross-sectional survey revealed an increase of 1.52 CONSORT items (0–10 scale, 95% CI 1.05 to 2.0) in articles conforming to a subheading system such as the one proposed in our study. Additionally, another RCT showed a difference of 1.78 CONSORT items (0–10 scale, 95% CI 0.39 to 3.23) between the manuscripts that received an additional review by a reporting guideline expert focused on 8 core CONSORT items and those that underwent usual peer review [12]. However, other strategies focused on the manuscript submission, peer review, and manuscript revision stages have been proven unsuccessful: requesting authors to submit a checklist together with the manuscripts [9], asking standard peer reviewers to check specific reporting guideline items [3], and implementing a web-based tool at the manuscript revision stage [27].

Regarding the reporting quality of each item, our results match those of previous studies that have shown remarkable improvements in key methodological items that are common to SPIRIT and CONSORT and that are usually poorly reported in RCT reports: *outcomes, blinding, or allocation concealment mechanism* [12]. For this reason, helping authors properly report these items in RCT protocols could have a remarkable impact on the reporting quality of the final RCT reports. Notably, it is surprising that in both periods, there was a low proportion of manuscripts (47% and 53%, respectively) that correctly reported the item *Outcomes*. This was mainly due to the lack of explicit differentiation between the primary and secondary outcomes and the failure to include their specific measurement variables. Additionally, less than half of the manuscripts in the post-intervention period (47%, 8 of 17) and none in the pre-intervention period included an adequate description of item 22 (*Harms*). We hypothesize that the reason for this is that, unlike many medical or pharmacological RCTs, most physiotherapy interventions are not considered potentially harmful. This could make authors less prone to report the absence or presence of harms, even though SPIRIT guidelines indicate so.

### Strengths and limitations

The strengths of the study include that the intervention was implemented in a real setting with no disruption to usual procedures in the master’s program. Also, the intervention evaluated has no cost, and it could be easily implemented in different contexts (journals, ethics committees, or education) and for other reporting guidelines. In addition, the study outcomes were assessed in duplicate by two blinded assessors.

We also mention several limitations. First, we did not use an RCT design, which may have affected the validity of the study results. For this reason, factors other than the intervention might have influenced the completeness of reporting of the RCT protocols included in the study. For example, even though the baseline characteristics of the protocols were similar across the two periods, the characteristics of the students developing the protocols might have been different. However, our results are similar to those of previous studies that evaluated other interventions focused on the CONSORT guidelines [12, 13]. Second, our participants were master’s degree students and most of them were not familiar with the task of writing RCT protocols. For this reason, these students could have benefitted more from the use of the templates than more experienced researchers. This hampers the generalisability of our findings to researchers with more experience as the real effect of the intervention might be smaller for that population. Also, the 3-h instruction on the SPIRIT items that was common to the two periods could not be as useful for them as it was for more inexperienced researchers. Third, we included study protocols from only one master’s program in the field of physiotherapy. Furthermore, our intervention focused only on 10 items of the SPIRIT guidelines, and the results could be different if the whole checklist or other guidelines were considered. Fourth, the study was not pre-registered, but we have made available the study protocol that was submitted and approved by the ethics committee [23]. Finally, there is no validated outcome measure that evaluates the completeness of reporting of research manuscripts. For this reason, we used the SPIRIT checklist, which is not intended to be an evaluation tool but rather just guidance for reporting [28]. However, this decision is consistent with the evaluation strategy of previous research in this field.

### Implications

This is the first intervention focused on the protocol writing stage that has ever been evaluated as to whether it improves the completeness of reporting. Furthermore, this is also the first intervention that consists of training biomedical students on the practical use of reporting guidelines [7]. The results shown here should stimulate the implementation of this and other research-based educational interventions to help students acquire competencies regarding research reporting and methodology [20, 21]. Some facilitators of this strategy are that it has no cost, it would be easy to implement in different contexts (e.g., education, ethics boards, or journals), and it could be followed for other reporting guidelines. Interestingly, future research should evaluate, preferably using an RCT design, whether similar benefits can be obtained 1) for other common reporting guidelines, such as CONSORT, STROBE or PRISMA, 2) in other contexts, such as ethics boards or journals, and 3) for other populations, such as more experienced researchers.

Improving adherence to SPIRIT guidelines is fundamental for different reasons. First, it makes RCT protocols more transparent and complete, allowing readers to fully understand the rationale, methods, and ethical aspects of RCTs. Second, as the background and methods sections of CONSORT are very similar to those of SPIRIT, improving adherence to SPIRIT makes it easier to comply with CONSORT requirements. Finally, even though SPIRIT provides reporting rather than methodological guidance, using SPIRIT makes authors aware of certain methodological aspects that they need to consider when carrying out an RCT, which can improve the study’s conduct.

## Conclusions

This study provides evidence that the use of templates for RCT protocols tailored to the SPIRIT guidelines improves the completeness of reporting of RCT protocols. This strategy could be applied to other reporting guidelines and enforced by biomedical journals, ethics boards, and universities to help improve the completeness of reporting of biomedical research.

### Supplementary Information

Additional file 1: SPIRIT-tailored template for RCT protocols (English version).

Additional file 2: Rules for the assessment of certain SPIRIT items.

Additional file 3: R script for the data analysis.

## Data Availability

We collected no personal data from the students who developed the manuscripts. We have made publicly available the censored version of all included RCT protocols and the dataset of the duplicate assessment of the SPIRIT items [23].

## Abbreviations

CONSORT: Consolidated Standards of Reporting Trials
RCT: Randomized Controlled Trial
SPIRIT: Standard Protocol Items: Recommendations for Interventional Trials
