# Supplementary material for: Enhancing reporting through structure: a before and after study on the effectiveness of SPIRIT-based templates to improve the completeness of reporting of randomized controlled trial protocols
Source: Res Integr Peer Rev. 2024 May 31;9:6. doi: 10.1186/s41073-024-00147-7 (PMC11140857; doi:10.1186/s41073-024-00147-7)
Supplement: Supplementary file 1 — Additional file 1: SPIRIT-tailored template for RCT protocols (English version). [file 41073_2024_147_MOESM1_ESM.docx]

The numbers in curly brackets (e.g. {5a}) are [SPIRIT](https://www.bmj.com/content/bmj/346/bmj.e7586.full.pdf) item identifiers. **You can find examples of how to report each item in the** [**SPIRIT 2013 explanation and elaboration document**](https://www.bmj.com/content/bmj/346/bmj.e7586.full.pdf) (Chan et al. 2013).

**Title {1}**

SPIRIT guidance: Descriptive title identifying the study design, population, interventions, and, if applicable, trial acronym.

**Abstract**

The Abstract should not exceed 350 words. Please minimize the use of abbreviations and do not cite references in the abstract. The abstract must include the following separate sections (in separate paragraphs):

**Background**: The context of the study. Please, include the specific objectives and hypothesis.

**Methods**: How the study will be performed. Please, include the following information:

1. Participants (eligibility criteria for participants and the settings where the data will be collected)
2. Interventions (Interventions intended for each group)
3. Outcomes (Clearly defined primary outcome for this report)
4. Randomisation (How participants will be allocated to interventions)
5. Blinding (Whether participants, care givers, and those assessing the outcomes were blinded to group assignment)

**Discussion**: Potential implications.

**List of contents**

**List of abbreviations**

**List of tables**

**List of figures**

**Introduction**

**Background and rationale {6a}**

SPIRIT guidance: Description of research question and justification for undertaking the trial, including summary of relevant studies (published and unpublished) examining benefits and harms for each intervention.

**Hypotheses and objectives {7}**

SPIRIT guidance: Specific hypotheses or objectives, which should be divided into primary and secondary.

**Methods**

**Trial design {8}**

SPIRIT guidance: Description of trial design including type of trial (eg, parallel group, crossover, factorial, single group), allocation ratio, and framework (eg, superiority, equivalence, noninferiority, exploratory).

**Study setting {9}**

SPIRIT guidance: Description of study settings (eg, community clinic, academic hospital) and list of countries where data will be collected. Reference to where list of study sites can be obtained.

**Eligibility criteria {10}**

SPIRIT guidance: Inclusion and exclusion criteria for participants. If applicable, eligibility criteria for study centres and individuals who will perform the interventions (eg, surgeons, psychotherapists).

**Interventions {11a} [according to** [**TIDieR**](https://www.bmj.com/content/bmj/348/bmj.g1687.full.pdf) **guidelines]**
SPIRIT guidance: Interventions for each group with sufficient detail to allow replication, including how and when they will be administered. Please, report the items explained in [TIDieR guidance](https://www.bmj.com/content/bmj/348/bmj.g1687.full.pdf).

**Outcomes {12} and instruments {18a}**

SPIRIT guidance {12}: Primary, secondary, and other outcomes, including the specific measurement variable (eg, systolic blood pressure), analysis metric (eg, change from baseline, final value, time to event), method of aggregation (eg, median, proportion), and time point for each outcome. Explanation of the clinical relevance of chosen efficacy and harm outcomes is strongly recommended.

SPIRIT guidance {18a}: Plans for assessment and collection of outcome, baseline, and other trial data, including any related processes to promote data quality (eg, duplicate measurements, training of assessors) and a description of study instruments (eg, questionnaires, laboratory tests) along with their reliability and validity, if known. Reference to where data collection forms can be found, if not in the protocol.

**Participant timeline {13}**

SPIRIT guidance: Time schedule of enrolment, interventions (including any run-ins and washouts), assessments, and visits for participants. A schematic diagram is highly recommended (see figure at <http://www.spirit-statement.org/publications-downloads/>).

**Sample size {14}**

SPIRIT guidance: Estimated number of participants needed to achieve study objectives and how it was determined, including clinical and statistical assumptions supporting any sample size calculations.

**Recruitment {15}**

SPIRIT guidance: Strategies for achieving adequate participant enrolment to reach target sample size.

**Allocation of interventions: sequence generation, concealment mechanism and implementation {16a, 16b y 16c}**

SPIRIT guidance (16a): Method of generating the allocation sequence (eg, computer-generated random numbers), and list of any factors for stratification. To reduce predictability of a random sequence, details of any planned restriction (eg, blocking) should be provided in a separate document that is unavailable to those who enroll participants or assign interventions.

SPIRIT guidance (16b): Mechanism of implementing the allocation sequence (eg, central telephone; sequentially numbered, opaque, sealed envelopes), describing any steps to conceal the sequence until interventions are assigned.

SPIRIT guidance (16c): Who will generate the allocation sequence, who will enroll participants, and who will assign participants to interventions.

**Blinding {17a}**

SPIRIT guidance: Who will be blinded after assignment to interventions (eg, trial participants, care providers, outcome assessors, data analysts), and how.

**Statistical methods for primary and secondary outcomes {20a}**

SPIRIT guidance: Statistical methods for analysing primary and secondary outcomes. Methods for any additional analyses (eg, subgroup and adjusted analyses).

**Ethical considerations:**

**Confidentiality {27}**

SPIRIT guidance: How personal information about potential and enrolled participants will be collected, shared, and maintained in order to protect confidentiality before, during, and after the trial.

**Informed consent {26a}**

SPIRIT guidance: Who will obtain informed consent or assent from potential trial participants or authorised surrogates, and how.

**Adverse event reporting and harms {22}**

SPIRIT guidance: Plans for collecting, assessing, reporting, and managing solicited and spontaneously reported adverse events and other unintended effects of trial interventions or trial conduct.

**Discussion**

Possible implications and projection of the study.

**[OPTIONAL] Pilot test of instruments and data collection**

If a pilot test of the instruments and data collection has been performed, describe it. Present 1) a table with the characteristics of the subjects included, 2) the problems encountered in the test of instruments and data collection, and 3) a table with the data obtained for each subject, without doing any statistical analysis.

**References**

Examples of the Vancouver reference style are shown below.

**Example reference style:**

*Article within a journal*

Smith JJ. The world of science. Am J Sci. 1999;36:234-5.

*Article within a journal (no page numbers)*

Rohrmann S, Overvad K, Bueno-de-Mesquita HB, Jakobsen MU, Egeberg R, Tjønneland A, et al. Meat consumption and mortality - results from the European Prospective Investigation into Cancer and Nutrition. BMC Medicine. 2013;11:63.

*Article within a journal by DOI*

Slifka MK, Whitton JL. Clinical implications of dysregulated cytokine production. Dig J Mol Med. 2000; doi:10.1007/s801090000086.

*Article within a journal supplement*

Frumin AM, Nussbaum J, Esposito M. Functional asplenia: demonstration of splenic activity by bone marrow scan. Blood 1979;59 Suppl 1:26-32.

*Book chapter, or an article within a book*

Wyllie AH, Kerr JFR, Currie AR. Cell death: the significance of apoptosis. In: Bourne GH, Danielli JF, Jeon KW, editors. International review of cytology. London: Academic; 1980. p. 251-306.

*OnlineFirst chapter in a series (without a volume designation but with a DOI)*

Saito Y, Hyuga H. Rate equation approaches to amplification of enantiomeric excess and chiral symmetry breaking. Top Curr Chem. 2007. doi:10.1007/128_2006_108.

*Complete book, authored*

Blenkinsopp A, Paxton P. Symptoms in the pharmacy: a guide to the management of common illness. 3rd ed. Oxford: Blackwell Science; 1998.

*Online document*

Doe J. Title of subordinate document. In: The dictionary of substances and their effects. Royal Society of Chemistry. 1999. http://www.rsc.org/dose/title of subordinate document. Accessed 15 Jan 1999.

*Online database*

Healthwise Knowledgebase. US Pharmacopeia, Rockville. 1998. http://www.healthwise.org. Accessed 21 Sept 1998.

*Supplementary material/private homepage*

Doe J. Title of supplementary material. 2000. http://www.privatehomepage.com. Accessed 22 Feb 2000.

*University site*

Doe, J: Title of preprint. http://www.uni-heidelberg.de/mydata.html (1999). Accessed 25 Dec 1999.

*FTP site*

Doe, J: Trivial HTTP, RFC2169. ftp://ftp.isi.edu/in-notes/rfc2169.txt (1999). Accessed 12 Nov 1999.

*Organization site*

ISSN International Centre: The ISSN register. http://www.issn.org (2006). Accessed 20 Feb 2007.

*Dataset with persistent identifier*

Zheng L-Y, Guo X-S, He B, Sun L-J, Peng Y, Dong S-S, et al. Genome data from sweet and grain sorghum (Sorghum bicolor). GigaScience Database. 2011. <http://dx.doi.org/10.5524/100012>.
