# Supplementary material for: Enhancing reporting through structure: a before and after study on the effectiveness of SPIRIT-based templates to improve the completeness of reporting of randomized controlled trial protocols
Source: Res Integr Peer Rev. 2024 May 31;9:6. doi: 10.1186/s41073-024-00147-7 (PMC11140857; doi:10.1186/s41073-024-00147-7)
Supplement: Supplementary file 2 — Additional file 2: Rules for the assessment of certain SPIRIT items. [file 41073_2024_147_MOESM2_ESM.docx]

Rules about specific items:

- **Item 12** (“*Primary, secondary, and other outcomes, including the specific measurement variable (eg, systolic blood pressure), analysis metric (eg, change from baseline, final value, time to event), method of aggregation (eg, median, proportion), and time point for each outcome. Explanation of the clinical relevance of chosen efficacy and harm outcomes is strongly recommended*”): Inadequately reported if, even though all the corresponding information was reported for the primary outcome, there was missing information for one or more secondary outcomes.
- **Item 17a** (“*Who will be blinded after assignment to interventions (eg, trial participants, care providers, outcome assessors, data analysts) and how*”): adequately reported if blinding was not performed and authors explicitly said so, and inadequately reported if blinding was assumed to be not performed and authors did not mention it in the manuscript. Also, inadequately reported if authors report that blinding was performed but not how it was implemented.
