# Supplementary material for: Enhancing reporting through structure: a before and after study on the effectiveness of SPIRIT-based templates to improve the completeness of reporting of randomized controlled trial protocols
Source: Res Integr Peer Rev. 2024 May 31;9:6. doi: 10.1186/s41073-024-00147-7 (PMC11140857; doi:10.1186/s41073-024-00147-7)
Supplement: Supplementary file 3 — Additional file 3: R script for the data analysis. [file 41073_2024_147_MOESM3_ESM.docx]

# Loading the data

library("readxl")

data <- read_excel("Scores.xlsx")

# Descriptive statistics per group

data_bef <- subset(data, data$Period == "Before")

mean(data_bef$Score)

sd(data_bef$Score)

data_aft <- subset(data, data$Period == "After")

mean(data_aft$Score)

sd(data_aft$Score)

# Primary outcome analysis

model <- lm(data$Score ~ data$Period + data$Call + data$Language)

summary(model)

confint(model)

plot(model) # checking assumptions

# Confidence Interval with Bootstrapping

na <- 17

nb <- 17

nboot <- 10000

set.seed(1111)

diff.mean1 <- c()

for (k in 1:nboot){

sel <- sample(1:(na + nb), na + nb, rep=TRUE)

reporting.boot <- data[sel, ]

diff.mean1[k] <- coefficients(lm(reporting.boot$Score ~ reporting.boot$Period + data$Call + data$Language

, reporting.boot))[2]

}

conf.int1 <- quantile(diff.mean1, c(0.025, 0.975), na.rm = TRUE)
